# Supplementary material for: A kinetically controlled platform for ligand-oligonucleotide transduction
Source: Nat Commun. 2021 Aug 2;12:4654. doi: 10.1038/s41467-021-24962-4 (PMC8329073; doi:10.1038/s41467-021-24962-4)
Supplement: Supplementary file 1 — Supplementary Information [file 41467_2021_24962_MOESM1_ESM.pdf]

Supplementary Information for

A Kinetically Controlled Platform for Ligand-Oligonucleotide Transduction

Qiu-Long Zhang, Liang-Liang Wang, Yan Liu, Jiao Lin, and Liang Xu*

MOE Key Laboratory of Bioinorganic and Synthetic Chemistry, School of Chemistry, Sun Yat-Sen University, Guangzhou, 510275, China.

**E-mail:* xuliang33@mail.sysu.edu.cn

Table of Contents

Supplementary Tables 3

Supplementary Table 1. 3

Supplementary Table 2. 4

Supplementary Table 3. 4

Supplementary Table 4. 5

Supplementary Table 5. 5

Supplementary Table 6. 6

Supplementary Table 7. 6

Supplementary Table 8. 6

Supplementary Table 9. 7

Supplementary Table 10. 7

Supplementary Table 11............ 8

Supplementary Table 12. 8

Supplementary Table 13. 9

Supplementary Table 14. 9

Supplementary Figures 11

Supplementary Fig. 1. 11

Supplementary Fig. 2. 12

Supplementary Fig. 3. 12

Supplementary Fig. 4. 13
Supplementary Fig. 5. 14
Supplementary Fig. 6. 15
Supplementary Fig. 7. 16
Supplementary Fig. 8. 17
Supplementary Fig. 9. 18
Supplementary Fig. 10. 18
Supplementary Fig. 11. 19
Supplementary Fig. 12. 20
Supplementary Fig. 13. 21
Supplementary Fig. 14. 22
Supplementary Fig. 15. 23
Supplementary Fig. 16. 24
Supplementary Fig. 17. 24
Supplementary Fig. 18. 25
Supplementary Fig. 19. 25
Supplementary Fig. 20. 26
Supplementary Fig. 21. 26
Supplementary Fig. 22. 27

Supplementary Tables

Supplementary Table 1. DNA sequences for optimization of the toehold length (related to Supplementary Fig. 1).

Strand name	5'-Sequence-3’
Hp-7nt	GTCAACG TT CGAGTGATCTATATAAGATCACTCG TTG GATAGGTAGGGTACGCT
Hp-8nt	TGTCAACG TT CGAGTGATCTATATAAGATCACTCG TTG GATAGGTAGGGTACGCT
Hp-9nt	ATGTCAACG TT CGAGTGATCTATATAAGATCACTCG TTG GATAGGTAGGGTACGCT
Hp-10nt	GATGTCAACG TT CGAGTGATCTATATAAGATCACTCG TTG GATAGGTAGGGTACGCT
Hp-11nt	TGATGTCAACG TT CGAGTGATCTATATAAGATCACTCG TTG GATAGGTAGGGTACGCT
Hp-12nt	GTGATGTCAACG TT CGAGTGATCTATATAAGATCACTCG TTG GATAGGTAGGGTACGCT
Loop-7nt	GTCAACG TT TGCGTCGAGGTTATAATGCCATACT TTG GATAGGTAGGGTACGCT
Loop-8nt	TGTCAACG TT TGCGTCGAGGTTATAATGCCATACT TTG GATAGGTAGGGTACGCT
Loop-9nt	ATGTCAACG TT TGCGTCGAGGTTATAATGCCATACT TTG GATAGGTAGGGTACGCT
Loop-10nt	GATGTCAACG TT TGCGTCGAGGTTATAATGCCATACT TTG GATAGGTAGGGTACGCT
Loop-11nt	TGATGTCAACG TT TGCGTCGAGGTTATAATGCCATACT TTG GATAGGTAGGGTACGCT
Loop-12nt	GTGATGTCAACG TT TGCGTCGAGGTTATAATGCCATACT TTG GATAGGTAGGGTACGCT

Supplementary Table 2. DNA sequences for optimization of the stem design in the ATP aptamer (related to Figure 2).

Strand name	5'-Sequence-3’
Apt-ATP-4bp-ACCT	GCAATCTCT TT ACCTGGGGGAGTATTGCGGAGGAAGGT TTG CCATGACCAGTAGTCTC
Apt-ATP-3bp-CCT	GCAATCTCT TT CCTGGGGGAGTATTGCGGAGGAAGG TTG CCATGACCAGTAGTCTC
Apt-ATP-3bp-CTT	GCAATCTCT TT CTTGGGGGAGTATTGCGGAGGAAAG TTG CCATGACCAGTAGTCTC
Apt-ATP-3bp-TCT	GCAATCTCT TT TCTGGGGGAGTATTGCGGAGGAAGA TTG CCATGACCAGTAGTCTC
Apt-ATP-2bp-CT	GCAATCTCT TT CTGGGGGAGTATTGCGGAGGAAG TTG CCATGACCAGTAGTCTC
Apt-ATP-1bp-T	GCAATCTCT TT TGGGGGAGTATTGCGGAGGAA TTG CCATGACCAGTAGTCTC

Supplementary Table 3. DNA sequences for optimization of the stem design in the Ochratoxin A (OTA) aptamer (related to Figure 2 and Supplementary Fig. 4).

Strand name	5'-Sequence-3’
Apt-OTA-4bp	ATGTCAACG TT AGCTTGATCGGGTGTGGGTGGCGTAAAGGGAGCATCGGACAGCT TTG GATAGGTAGGGTACGCT
Apt-OTA-3bp	ATGTCAACG TT ACTTGATCGGGTGTGGGTGGCGTAAAGGGAGCATCGGACAGT TTG GATAGGTAGGGTACGCT
Apt-OTA-2bp	ATGTCAACG TT CTTGATCGGGTGTGGGTGGCGTAAAGGGAGCATCGGACAG TTG GATAGGTAGGGTACGCT

Supplementary Table 4. DNA sequences for optimization of the stem design in the L-tyrosinamide (Tym) aptamer (related to Figure 2 and Supplementary Fig. 6)

Strand name	5'-Sequence-3’
Apt- L-Tym -4bp	GCAATCTCT TT GGTTTTGTGGTGTGTGAGTGCGGTGCCCTAACC TTG CCATGACCAGTAGTCTC
Apt- L-Tym -3bp	GCAATCTCT TT GTTTTGTGGTGTGTGAGTGCGGTGCCCTAAC TTG CCATGACCAGTAGTCTC
Apt- L-Tym -2bp	GCAATCTCT TT GTTTGTGGTGTGTGAGTGCGGTGCCCTAC TTG CCATGACCAGTAGTCTC
Apt- L-Tym -1bp	GCAATCTCT TT GTTGTGGTGTGTGAGTGCGGTGCCCTC TTG CCATGACCAGTAGTCTC

Supplementary Table 5. DNA sequences for optimization of the stem design in the thrombin aptamer (related to Figure 3 and Supplementary Fig. 8).

Strand name	5'-Sequence-3’
Apt-Thr-4bp	ATGTCAACG T AGTCCGTGGTAGGGCAGGTTGGGGTGACT TG GATAGGTAGGGTACGCT
Apt-Thr-3bp	ATGTCAACG TT GTCCGTGGTAGGGCAGGTTGGGGTGAC TTG GATAGGTAGGGTACGCT
Apt-Thr-2bp	ATGTCAACG TT TCCGTGGTAGGGCAGGTTGGGGTGA TTG GATAGGTAGGGTACGCT
Apt-Thr-1bp	ATGTCAACG TT CCGTGGTAGGGCAGGTTGGGGTG TTG GATAGGTAGGGTACGCT
Apt-Thr-0bp	ATGTCAACG TT CGTGGTAGGGCAGGTTGGGGT TTG GATAGGTAGGGTACGCT

Supplementary Table 6. DNA sequences for optimization of the stem design in the PDGF A aptamer (related to Figure 3 and Supplementary Fig. 11).

Strand name	5'-Sequence-3'
Apt-PDGF-3bp	ATGTCAACG TT AGGCTACGGCACGTAGAGCATCACCATGATCCT TTG GATAGGTAGGGTACGCT
Apt-PDGF-2bp	ATGTCAACG TT GGCTACGGCACGTAGAGCATCACCATGATCC TTG GATAGGTAGGGTACGCT
Apt-PDGF-1bp	ATGTCAACG TT GCTACGGCACGTAGAGCATCACCATGATC TTG GATAGGTAGGGTACGCT

Supplementary Table 7. DNA sequences utilized in the logic operations between ATP and thrombin (related to Figure 4).

Strand name	5'-Sequence-3'
Apt-ATP-OR	ATGTCAACG TT CTTGGGGGAGTATTGCGGAGGAAAG TTG GATAGGTAGGGTACGCT
Apt-Thr-OR	ATGTCAACG TT GTCCGTGGTAGGGCAGGTTGGGGTGAC TTG GATAGGTAGGGTACGCT
Apt-ATP-NOT	TATCCAA CTTGGGGGAGTATTGCGGAGGAAAG AACGTTG
Apt-Thr-NOT	ATGTCAACG TT GTCCGTGGTAGGGCAGGTTGGGGTGAC TTG GATAGGTAGGGTACGCT
Apt-ATP-AND	CGCACGATCTTG CTTGGGGGAGTATTGCGGAGGAAAG TTG GATAGGTAGGGTACGCT
Apt-Thr-AND	GTGATGTCAACG TTGTCCGTGGTAGGGCAGGTTGGGGTGAC CAAGATCGTGCG

Supplementary Table 8. DNA sequences for optimization of the toehold length in the combinatory aptamer (related to Supplementary Fig. 13).

Strand name	5'-Sequence-3'
Apt-Thr-AND-9nt	ATGTCAACG TT GTCCGTGGTAGGGCAGGTTGGGGTGAC

	CAAGATCGTGCG
Apt-Thr-AND-10nt	GATGTCAACG TT GTCCGTGGTAGGGCAGGTTGGGGTGAC CAAGATCGTGCG
Apt-Thr-AND-11nt	TGATGTCAACG TTGTCCGTGGTAGGGCAGGTTGGGGTGAC CAAGATCGTGCG
Apt-Thr-AND-12nt	GTGATGTCAACG TTGTCCGTGGTAGGGCAGGTTGGGGTGAC CAAGATCGTGCG
Apt-ATP-AND	CGCACGATCTTG CTTGGGGGAGTATTGCGGAGGAAAG TTG GATAGGTAGGGTACGCT

Supplementary Table 9. DNA sequences utilized in the cascade reactions between ATP and thrombin (related to Figure 5).

Strand name	5'-Sequence-3’
Apt-Thr-A[a]	GATAGGTAGGGTACGCT GTT GTCCGTGGTAGGGCAGGTTGGGGTGAC TT AGTAACTCG
Apt-ATP-A[a]	ATGTCAACG TT CTTGGGGGAGTATTGCGGAGGAAAG TTG GATAGGTAGGGTACGCT
Masking strand A[a]	CGAGTTACTTTCAGCGTACCCTACCTATC
Apt-Thr-B[b]	TCATTGACG TT GTCCGTGGTAGGGCAGGTTGGGGTGAC TTG GTGCCAGTGCAATCTCT
Apt-ATP-B[b]	GTGCCAGTGCAATCTCT TT CTTGGGGGAGTATTGCGGAGGAAAG TTG CCATGACCAGTAGTCTC
Masking strand B[b]	AGAGATTGCACTGGCACCTTCGTCAATGA

[a] Sequences utilized in Figure 5a; [b] Sequences utilized in Figure 5b.

Supplementary Table 10. DNA sequences utilized in stimulating feedback (related to Supplementary Fig. 14)

Strand name	5'-Sequence-3’

Apt -Thr- stimulation	CTTGAAGCAGCTAACCGATACAGTTGCAGGTCCAG GTT GTCCGTGGTAGGGCAGGTTGGGGTGAC TT CGATAGAGA
Apt -ATP- stimulation	CTTGAAGTAAGCAGCGG GTT CTTGGGGGAGTATTGCGGAGGAAAG TT TACAGTTGCAGGTCCAG
Output-1-stimulation	CCATAGCCAGTAGTCTCCTTGAAGCAGCTAACCG
Complementary-1-stimulation (Strand *i*)	TCTCTATCGTTCCTGGACCTGCAACTGTATCGGTTAGCTGC TTCAAG
Output-2-stimulation	CCATAGCCAGTAGTCTCCTTGAAGTAAGCAGCGG
Complementary-2-stimulation (Strand *ii*)	GCAACTGTATTCCCGCTGCTTACTTCAAG

Supplementary Table 11. DNA sequences utilized in inhibiting feedback (related to Supplementary Fig. 15)

Strand name	5'-Sequence-3’
Apt- Thr-inhibition	CTTGAAGCAGCTAACCGATACAGTTGCAGGTCCAG GTT GTCCGTGGTAGGGCAGGTTGGGGTGAC TT CGATAGAGA
Apt -ATP- inhibition	GAGACTAGCAAGCGTGA GTT CTTGGGGGAGTATTGCGGAGGAAAG TT TACAGTTGCAGGTCCAG
Output-1-inhibition	CCATAGCCAGTAGTCTCCTTGAAGCAGCTAACCG
Complementary-1-inhibition (Strand *i*)	TCTCTATCGTTCCTGGACCTGCAACTGTATCGGTTAGCTGC TTCAAG
Output-2- inhibition	GCAAGCGTGACTTCAAGGAGACTA
Complementary-2-inhibition (Strand *ii*)	GCAACTGTATTCACTTGAAGTCACGCTTGC

Supplementary Table 12. DNA sequences utilized in the toehold exchange (TE) experiment (related to Figure 6a and Supplementary Fig. 19).

Strand name	5'-Sequence-3’
Apt-ATP-TE	CAATCTCT TT CTTGGGGGAGTATTGCGGAGGAAAG TTG CCATAGCCAGTAGTCTC
Apt-Thr-TE	CAATCTCT TT GTCCGTGGTAGGGCAGGTTGGGGTGAC TTG CCATAGCCAGTAGTCTC
Apt-Output-TE	CCATAGCCAGTAGTCTCCTTGAAG
Apt-Complementary-TE	GAGACTACTGGCTATGGCTTAGAGATTGCC
TE-Displacement (Strand *i*)	CTCACTGCAGGTTATCCCAGGATTCCATAGCCAGTAGTCTC
TE-Complementary (Strand *ii*)	CTTCAAGGAGACTACTGGCTATGGAATCCTG
TE-Fuel (Strand *iii*)	CAGGATTCCATAGCCAGTAGTCTC

Supplementary Table 13. DNA sequences utilized in the DNAzyme experiment (related to Figure 6b and Supplementary Fig. 20).

Strand name	5'-Sequence-3’
Apt-ATP-DNAzyme	TAAGTTGC TT CTTGGGGGAGTATTGCGGAGGAAAG TTA TGAGATATCTCCGAGCC
Apt-Thr-DNAzyme	TAAGTTGC TT GTCCGTGGTAGGGCAGGTTGGGGTGAC TTA TGAGATATCTCCGAGCC
Apt-Output-DNAzyme	TGAGATATCTCCGAGCCGGACGAATACTCTTG
Apt-Complementary-DNAzyme	GGCTCGGAGATATCTCATTTGCAACTTAG
DNAzyme-Substrate	5’ BHQ1-CAAGAGTATrAGGATATCTC-FAM 3’

Supplementary Table 14. DNA sequences utilized in reporter systems.

Strand name	5’-Sequence-3’
Rep-1-FAM	5’(FAM)-GAGACTACTGGTCATGGCTTAGAGATTGCC

Rep-1-BHQ1	CCATGACCAGTAGTCTC-3’(BHQ1)
Rep-2-TMR	5’(TAMRA)-AGCGTACCCTACCTATCCTTCGTTGACATCAC
Rep-2-BHQ2	GATAGGTAGGGTACGCT-3’(BHQ2)
Rep-3-TMR	AATCCTGGGATAACCTGCAGTGAG-3’(TAMRA)
Rep-3-BHQ2	5’(BHQ2)-CTCACTGCAGGTTATCC
Rep-4-FAM	CTTCAAGGAGACTACTGGCTATGG-3’(FAM)
Rep-4-BHQ1	5’(BHQ1)-CCATAGCCAGTAGTCTC

Supplementary Figures

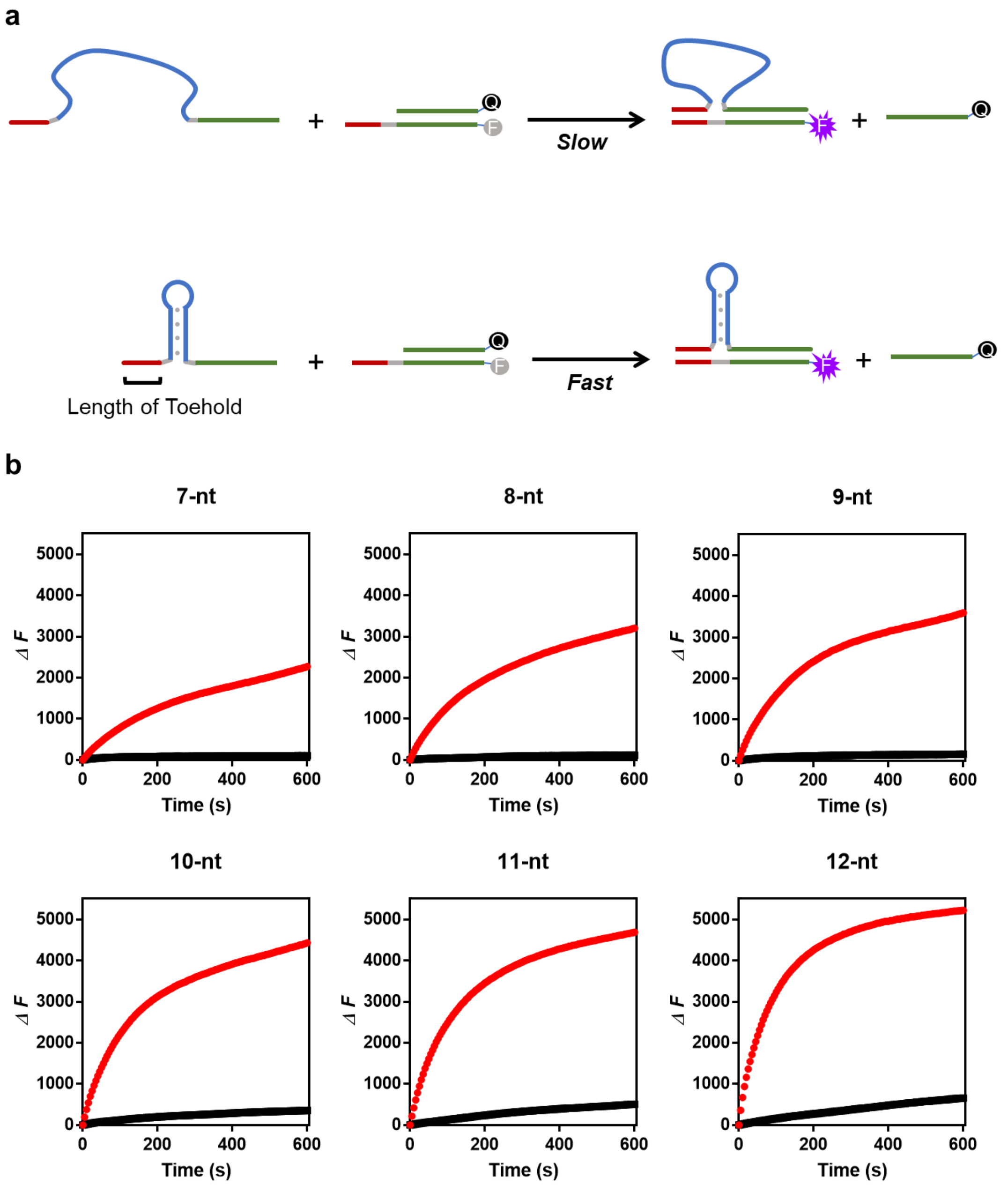

Supplementary Fig. 1. Examination of the kinetic control between the unstructured loop and the hairpin-based design. (**a**) Scheme for the designing concept. When the toehold and the displacement segment are separated by an unstructured loop, the strand displacement is very slow. In the contrast, if the toehold and the displacement segment are joined together by a defined duplex, the kinetic behavior of the strand invasion becomes very fast. (**b**) The length of the toehold can control the kinetic distinction between the two different designs. Kinetic behaviors with different lengths of toehold were measured and compared. The black dots

indicated the signals of the design with an unstructured loop; the red dots indicated the signals of the design with an hairpin structure. The Rep-2 system (25 nM) was utilized with the loop strand or the hairpin strand (50 nM) in this experiment. Sequence information related to this figure was listed in Supplementary Table 1. Source data are provided as a Source Data file.

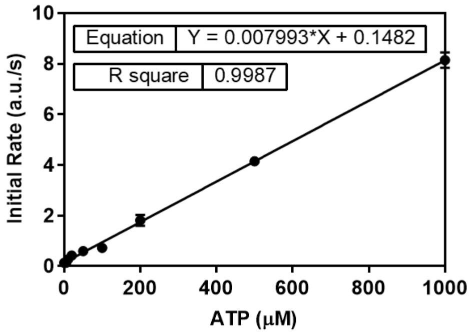

Supplementary Fig. 2. The linear fitting between the initial rates and the ATP concentrations (0-1000 μM) based on the data in Figure 2d. “a.u.”: arbitrary units for fluorescence. Data are presented as mean values with standard deviations (error bars) derived from three independent experiments. Those error bars shorter than the height of the symbol are not shown in the graph. The calculated LOD was ~7.6 μM. Source data are provided as a Source Data file.

a

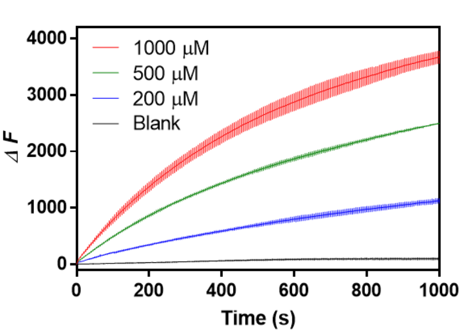

b

ATP	Kinetic rates in buffer	Kinetic rates in 10% serum	Recovery
1000 μM	8.14 ± 0.31	8.33 ± 0.71	102 ± 8%
500 μM	4.15 ± 0.02	4.92 ± 0.25	118 ± 5%
200 μM	1.81 ± 0.22	2.07 ± 0.23	114 ± 11%

Supplementary Fig. 3. Recovery test for detection of ATP in 10% serum samples. (a) Kinetic curves of fluorescence changes upon additions of different concentrations of ATP into 10% serum samples. (b) Comparison of initial kinetic rates in different samples. Data are presented as mean values with standard deviations (error bars) derived from three independent experiments. The recovery efficacy was ranged from 102% to 118%. Source data are provided as a Source Data file.

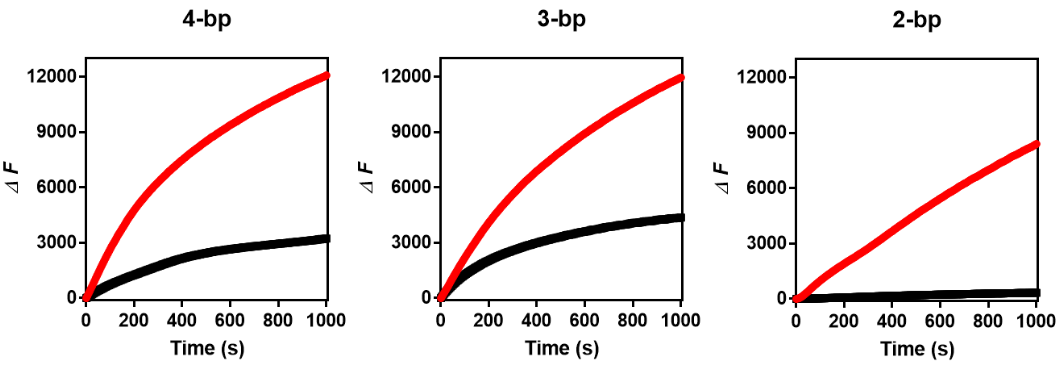

Supplementary Fig. 4. Optimization of the designed stem duplex for the transduction of mycotoxin ochratoxin A (OTA). Extra short stems were added into the design of the OTA aptamer. The kinetic control of the OTA-induced strand displacement reaction was performed based on the same design as depicted in Figure 2a. Different thermostabilities of these short duplex stem would govern the balance between the transduction efficiency and the background signal. The red dots indicated the kinetic data of the reporter system in the presence of 50 μM OTA. The black dots indicated the background signal without OTA. The Rep-2 system (25 nM) was utilized with the Apt-OTA strand (50 nM) in this experiment. Sequence information related to this figure was listed in Supplementary Table 3. Source data are provided as a Source Data file.

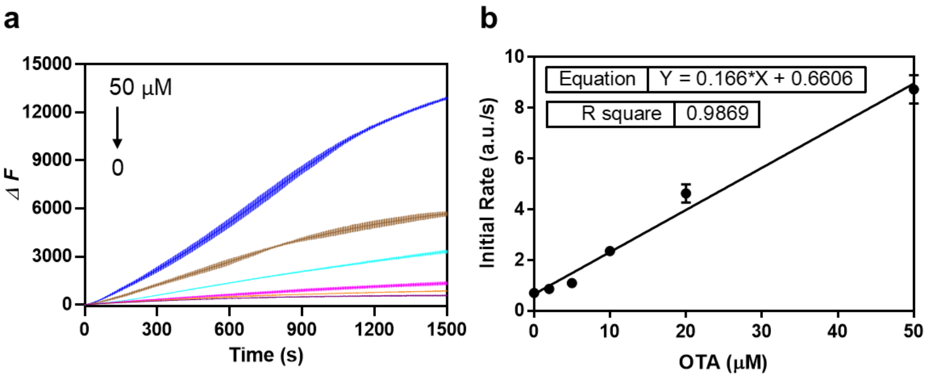

Supplementary Fig. 5. Concentration-dependent kinetic performance of the OTA-induced strand-displacement reaction based on the 2-bp design. (a) Kinetic curves of fluorescence changes upon additions of different concentrations of OTA. The concentrations of OTA were 50, 20, 10, 5, 2 and 0 μM, respectively. (b) The linear fitting between the initial rates and the OTA concentrations (0-50 μM). "a.u.": arbitrary units for fluorescence. Data are presented as mean values with standard deviations (error bars) derived from three independent experiments. Those error bars shorter than the height of the symbol are not shown in the graph. The calculated LOD was ~0.95 μM. Source data are provided as a Source Data file.

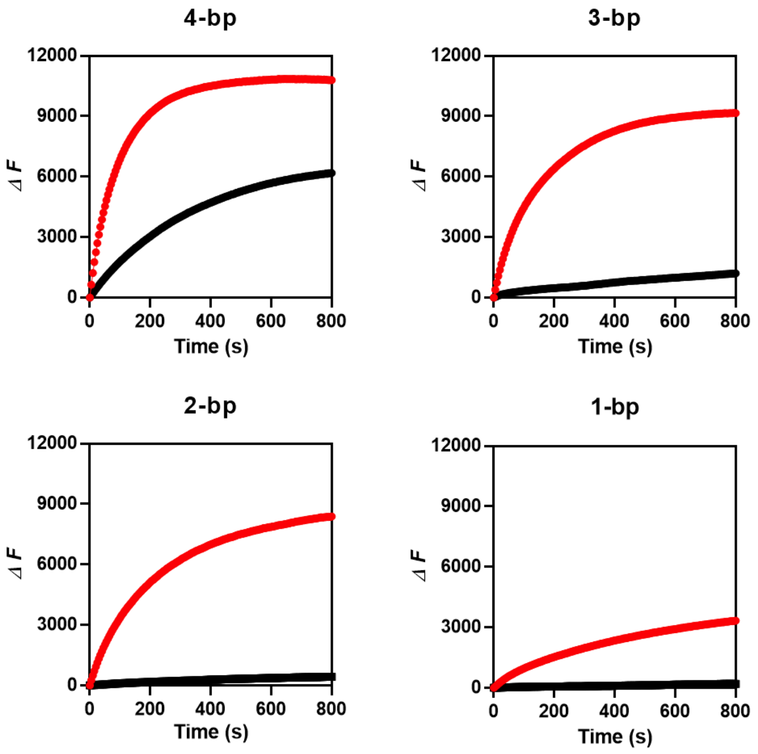

Supplementary Fig. 6. Optimization of the designed stem duplex for the transduction of L-tyrosinamide (Tym). Extra short stems were added into the design of the Tym aptamer. The kinetic control of the Tym-induced strand displacement reaction was performed based on the same design as depicted in Figure 2a. The red dots indicated the kinetic data of the reporter system in the presence of 100 μM Tym. The black dots indicated the background signal without Tym. Sequence information related to this figure was listed in Supplementary Table 4. Source data are provided as a Source Data file.

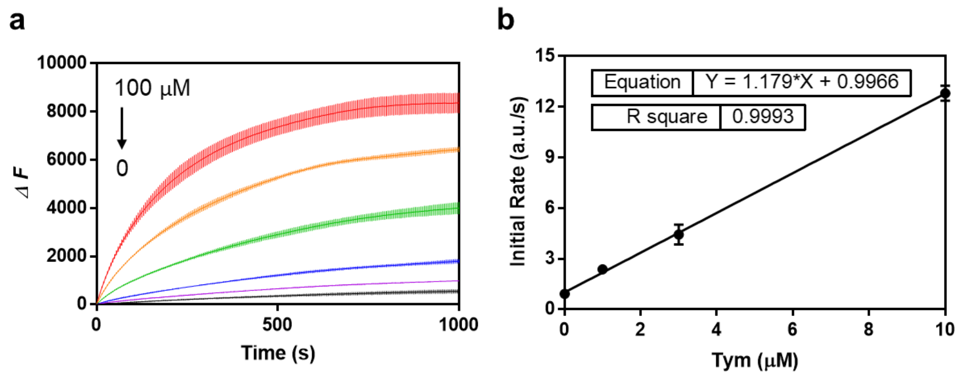

Supplementary Fig. 7. Concentration-dependent kinetic performance of the Tym-induced strand-displacement reaction based on the 2-bp design. (a) Kinetic curves of fluorescence changes upon additions of different concentrations of Tym. The concentrations of Tym were 100, 30, 10, 3, 1 and 0 μM, respectively. (b) The linear fitting between the initial rates and the Tym concentrations (0-10 μM). "a.u.": arbitrary units for fluorescence. Data are presented as mean values with standard deviations (error bars) derived from three independent experiments. Those error bars shorter than the height of the symbol are not shown in the graph. The calculated LOD was ~0.27 μM. Source data are provided as a Source Data file.

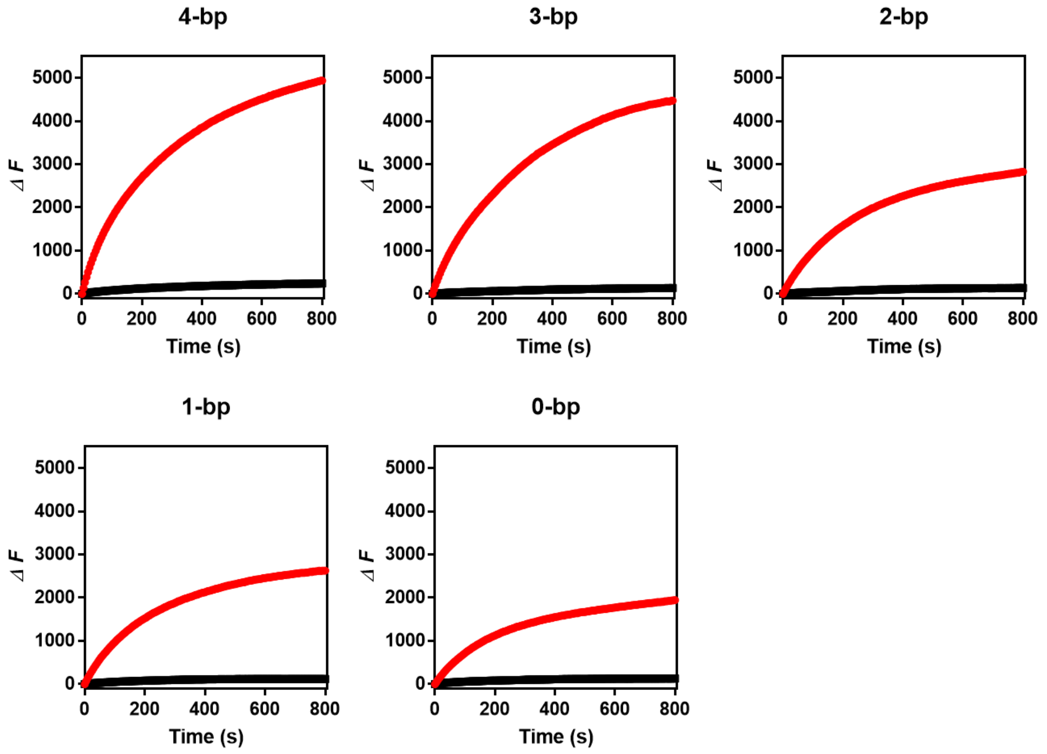

Supplementary Fig. 8. Optimization of the designed stem duplex for the transduction of thrombin. The kinetic control of the thrombin-induced strand displacement reaction was performed as depicted in Figure 3a. Different thermostabilities of these short duplex stem would govern the balance between the transduction efficiency and the background signal. The red dots indicated the kinetic data of the reporter system in the presence of 200 nM thrombin. The black dots indicated the background signal without thrombin. The Rep-2 system (25 nM) was utilized with the Apt-Thr strand (50 nM) in this experiment. Sequence information related to this figure was listed in Supplementary Table 5. Source data are provided as a Source Data file.

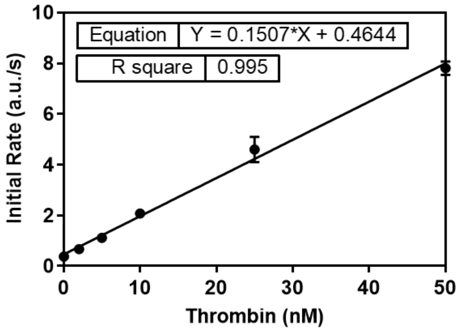

Supplementary Fig. 9. The linear fitting between the initial rates and the thrombin concentrations (0-50 nM) based on the data in Figure 3d. “a.u.”: arbitrary units for fluorescence. Data are presented as mean values with standard deviations (error bars) derived from three independent experiments. Those error bars shorter than the height of the symbol are not shown in the graph. The calculated LOD was ~0.96 nM. Source data are provided as a Source Data file.

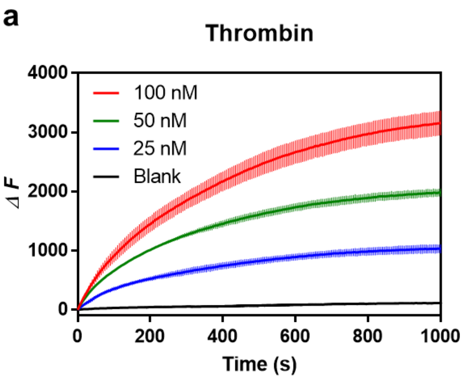

b

Thrombin	Kinetic rates in buffer	Kinetic rates in 1% serum	Recovery
100 nM	12.64 ±0.94	11.64 ± 1.51	92 ± 13%
50 nM	7.81 ± 0.26	8.38 ± 0.07	107 ± 1%
25 nM	4.60 ± 0.50	4.68± 1.00	102 ± 21%

Supplementary Fig. 10. Recovery test for detection of thrombin in 1% serum samples. (a) Kinetic curves of fluorescence changes upon additions of different concentrations of thrombin into 1% serum samples. (b) Comparison of initial kinetic rates in different samples. Data are presented as mean values with standard deviations (error bars) derived from three independent experiments. The recovery efficacy was ranged from 92% to 107%. Source data are provided as a Source Data file.

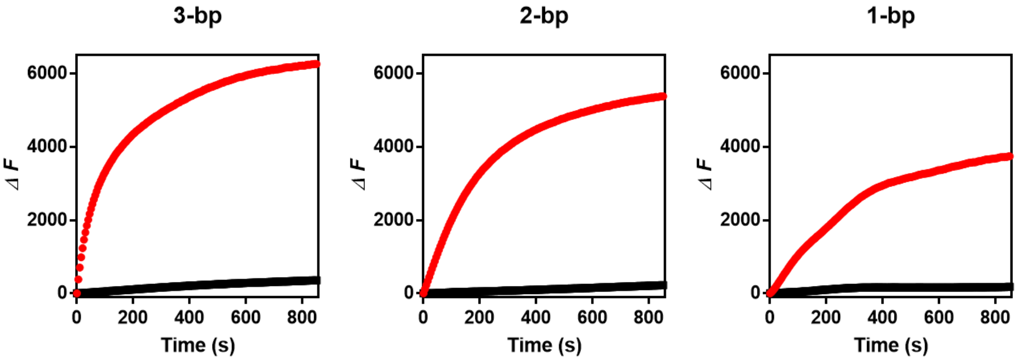

Supplementary Fig. 11. Optimization of the designed stem duplex for the transduction of PDGF. The kinetic control of the PDGF-induced strand displacement reaction was performed based on the same design as depicted in Figure 3a. Different thermostabilities of these short duplex stem would govern the balance between the transduction efficiency and the background signal. The red dots indicated the kinetic data of the reporter system in the presence of 50 nM PDGF. The black dots indicated the background signal without PDGF. The Rep-2 system (25 nM) was utilized with the Apt-PDGF strand (50 nM) in this experiment. Sequence information related to this figure was listed in Supplementary Table 6. Source data are provided as a Source Data file.

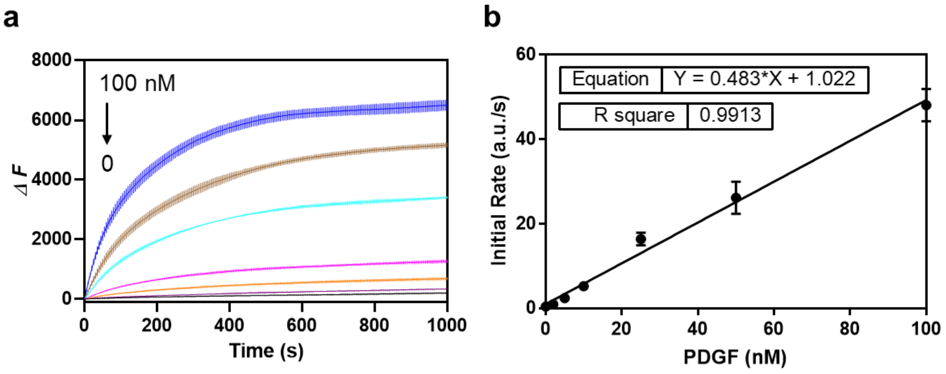

Supplementary Fig. 12. Concentration-dependent kinetic performance of the PDGF-induced strand-displacement reaction based on the 2-bp design. (a) Kinetic curves of fluorescence changes upon additions of different concentrations of PDGF. The concentrations of PDGF were 100, 50, 25, 10, 5, 2 and 0 nM, respectively. (b) The linear fitting between the initial rates and the PDGF concentrations (0-100 nM). "a.u.": arbitrary units for fluorescence. Data are presented as mean values with standard deviations (error bars) derived from three independent experiments. Those error bars shorter than the height of the symbol are not shown in the graph. The calculated LOD was ~0.73 nM. Source data are provided as a Source Data file.

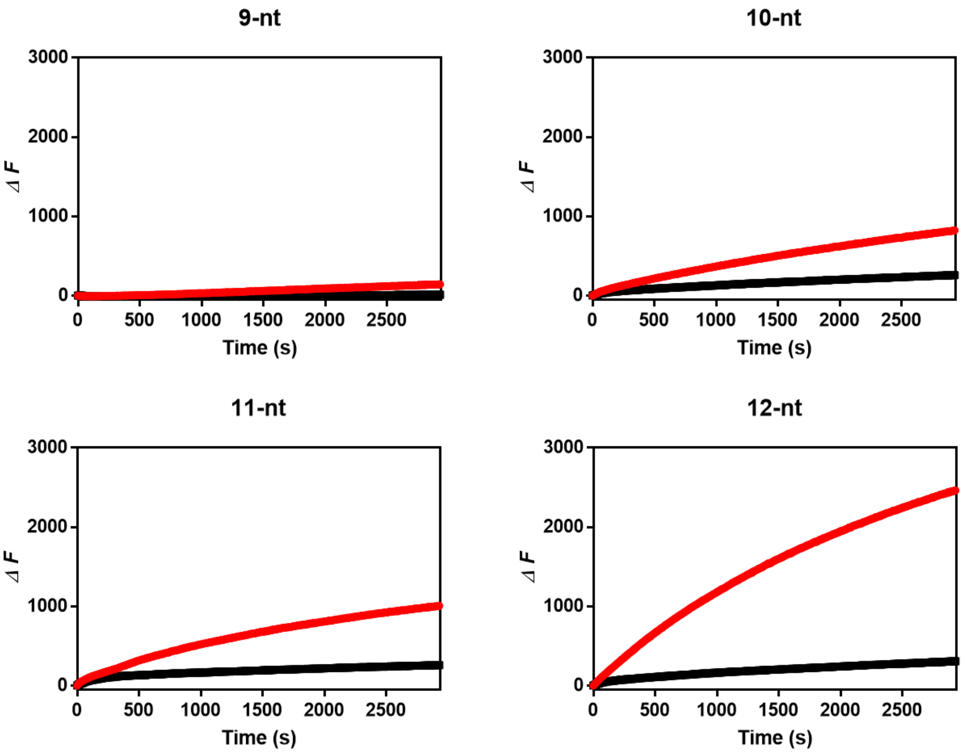

Supplementary Fig. 13. Optimization of the toehold length for the combinatory aptamer. Kinetic behaviors with different lengths of toeholds in the thrombin-ATP combinatory aptamer were examined based on the designing strategy depicted in Figure 4c. The kinetic data in presence of both 1 mM ATP and 200 nM thrombin (the red dots) and the background signal (the black dots) were measured and compared. The Rep-2 system was utilized in this experiment. Sequence information related to this figure was listed in Supplementary Table 8. Source data are provided as a Source Data file.

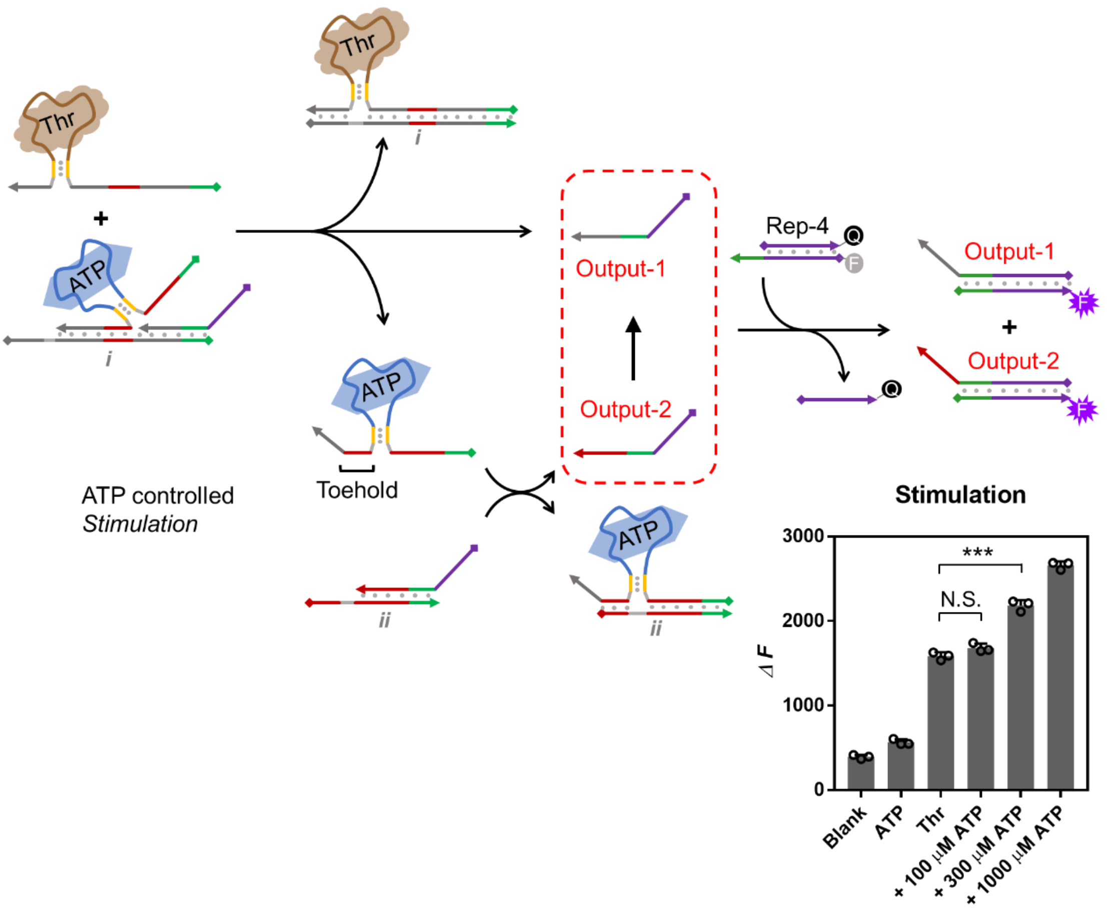

Supplementary Fig. 14. Stimulating feedback based on the cascade reaction. Thrombin can trigger the strand displacement to release the output strand 1 (Output-1) and the ATP-aptamer containing strand. With the presence of ATP, the ATP-controlled strand displacement can proceed upon the release of the ATP-aptamer containing strand to generate another output strand (Output-2). Both Output-1 and Output-2 can be detected by the reporter duplex (Rep-4). In this case, the Output-2 exhibited a stimulating effect on the signal reporting process. As observed in the data graph, thrombin (200 nM) can be clearly detected by the reporter duplex, but ATP alone (1 mM) could hardly produce strong signals compared with the blank control due to the masked aptamer-containing strand. However, addition of ATP into the thrombin (200 nM) system would greatly enhance the reporting signals. From another perspective, the signal of thrombin was significantly enhanced by ATP. Data are presented as mean values with standard deviations (error bars) derived from three independent experiments. “N.S.”: no significant difference; $***P = 0.0002$ (unpaired t test, two-tailed P value, n = 3). Sequence

information related to this figure was listed in Supplementary Table 10. Source data are provided as a Source Data file.

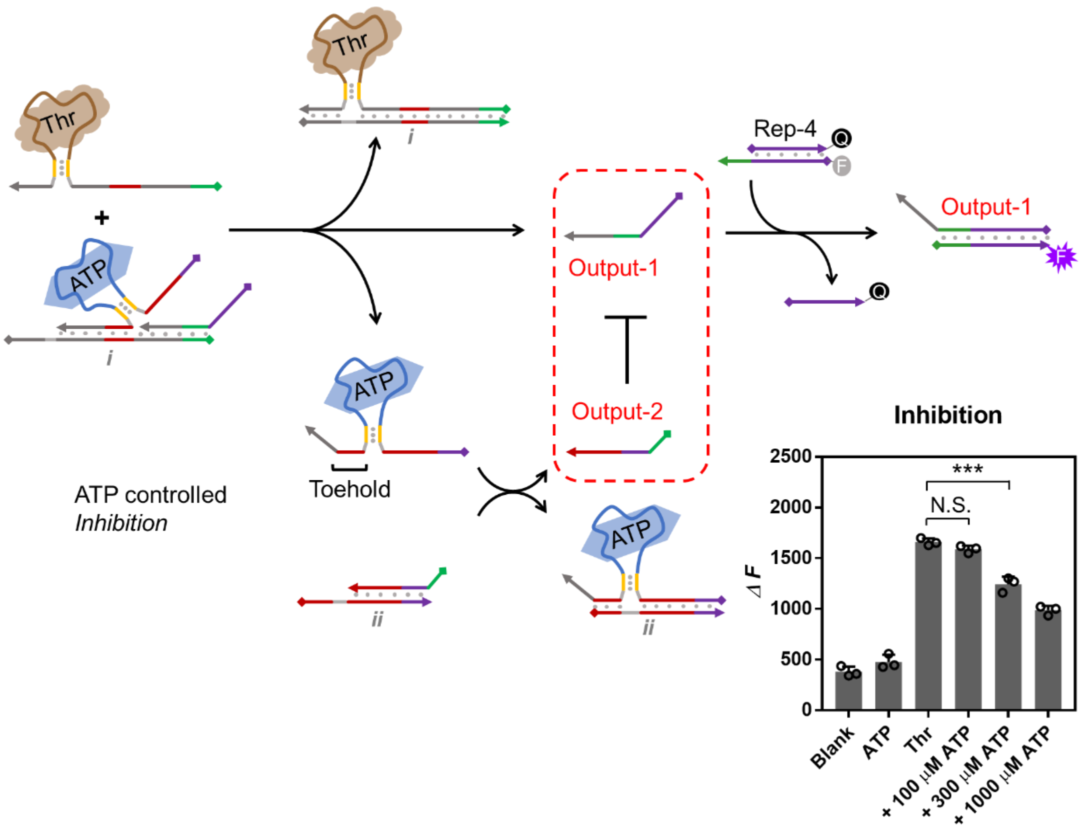

Supplementary Fig. 15. Inhibiting feedback based on the cascade reaction. Thrombin can trigger the strand displacement to release the output strand 1 (Output-1) and the ATP-aptamer containing strand. With the presence of ATP, the ATP-controlled strand displacement can proceed upon the release of the ATP-aptamer containing strand to generate another output strand (Output-2). The Output-2 is partially base-paired with the Output-1, and can in turn inhibit the reporting process of Output-1. As observed in the data graph, addition of ATP into the thrombin (200 nM) system would greatly reduce the reporting signals. Data are presented as mean values with standard deviations (error bars) derived from three independent experiments. "N.S.": no significant difference; ***P = 0.0009 (unpaired t test, two-tailed P value, n = 3). Sequence information related to this figure was listed in Supplementary Table 11. Source data are provided as a Source Data file.

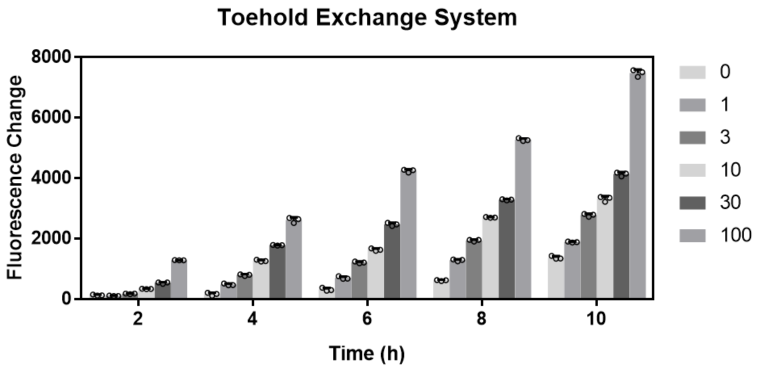

Supplementary Fig. 16. ATP-induced time-dependent fluorescence increase with incubation of the toehold exchange-based catalysis system. Concentrations of ATP were 0, 1, 3, 10, 30 and 100 μM, respectively. With the signal amplification by the toehold exchange system, even weak signals could be significantly enhanced after a long-time incubation. Data are presented as mean values with standard deviations (error bars) derived from three independent experiments. Source data are provided as a Source Data file.

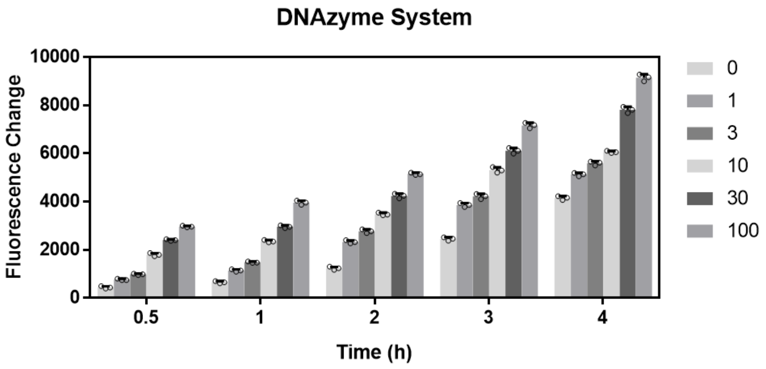

Supplementary Fig. 17. ATP-induced time-dependent fluorescence increase with incubation of the DNAzyme system. Concentrations of ATP were 0, 1, 3, 10, 30 and 100 μM, respectively. With the signal amplification by the DNAzyme system, even weak signals could be significantly enhanced after a long-time incubation. The catalysis of DNAzyme was relatively faster than the toehold exchange system. If the incubation time is too long, fluorescence difference between different concentrations of ATP would be reduced. Data are presented as mean values with standard deviations (error bars) derived from three independent experiments. Source data are provided as a Source Data file.

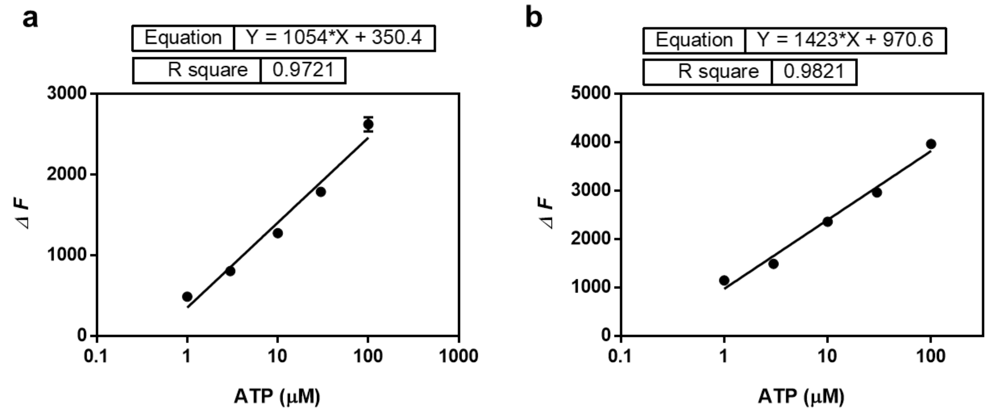

Supplementary Fig. 18. Fitting of ATP-induced fluorescence increase after 4-hr amplification in the toehold exchange system (a) or 1-hr amplification in the DNAzyme system (b). Signals were linearly fitted by plotting the x-axis on a log scale (1-100 μM). Data are presented as mean values with standard deviations (error bars) derived from three independent experiments. Those error bars shorter than the height of the symbol are not shown in the graph. LOD was defined as the lowest quantity of ATP that produces a fluorescence signal $3\sigma_b$ higher than the average level of the blank sample. The calculated LOD of ATP was ~0.93 μM in the toehold exchange system, and ~0.75 μM in the DNAzyme system. Source data are provided as a Source Data file.

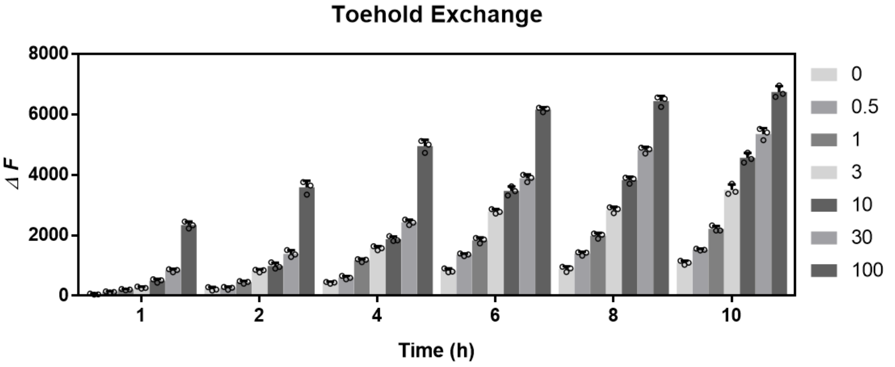

Supplementary Fig. 19. Thrombin-induced time-dependent fluorescence increase with incubation of the toehold exchange-based catalysis system. Concentrations of thrombin were 0, 0.5, 1, 3, 10, 30 and 100 nM, respectively. Data are presented as mean values with standard deviations (error bars) derived from three independent experiments. Sequence information related to this figure was listed in Supplementary Table 12. Source data are provided as a Source Data file.

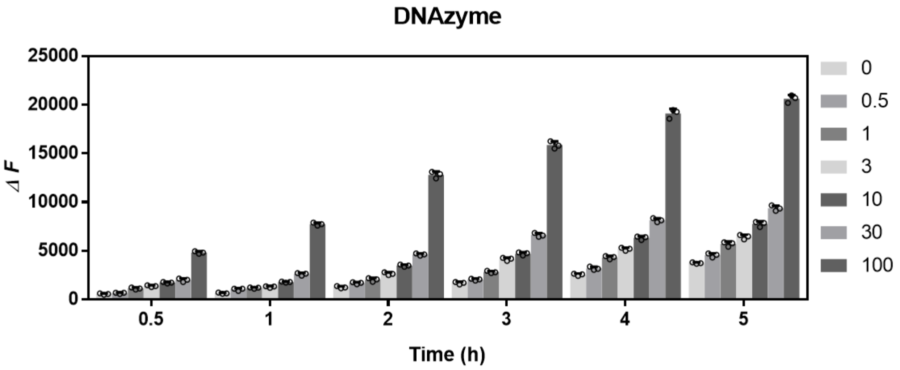

Supplementary Fig. 20. Thrombin-induced time-dependent fluorescence increase with incubation of the DNAzyme system. Concentrations of thrombin were 0, 0.5, 1, 3, 10, 30 and 100 nM, respectively. Data are presented as mean values with standard deviations (error bars) derived from three independent experiments. Sequence information related to this figure was listed in Supplementary Table 13. Source data are provided as a Source Data file.

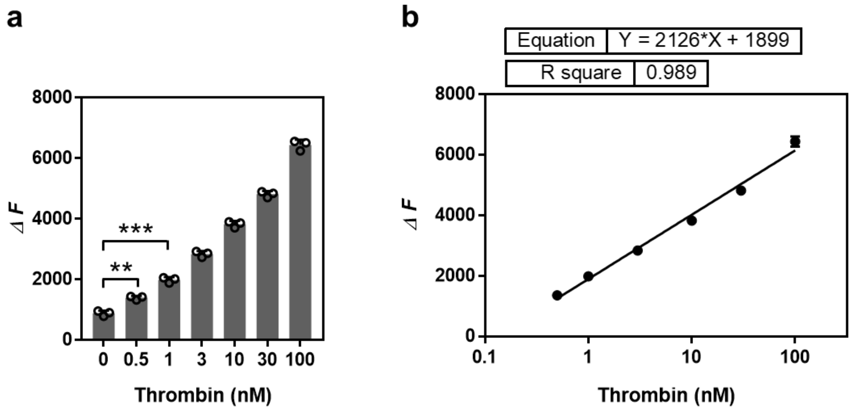

Supplementary Fig. 21. Quantification of thrombin-induced fluorescence change after 8-hr amplification in the toehold exchange system. (a) The fluorescence changes after 8-hour incubation in the presence of different concentrations of thrombin with the toehold exchange-based catalysis system. (b) Signals were linearly fitted by plotting the x-axis on a log scale (0.5-100 nM). Data are presented as mean values with standard deviations (error bars) derived from three independent experiments. Those error bars shorter than the height of the symbol are not shown in the graph. ***P* = 0.0012, ****P* = 0.0001 (unpaired t test, two-tailed *P* value, n = 3). LOD was defined as the lowest quantity of thrombin that produces a fluorescence signal $3\sigma_b$ higher than the average level of the blank sample. The calculated LOD of thrombin was ~0.35

nM. Source data are provided as a Source Data file.

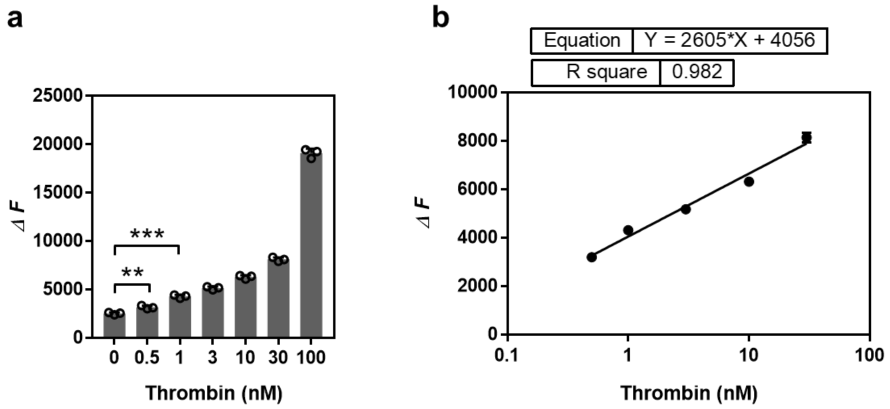

Supplementary Fig. 22. Quantification of thrombin-induced fluorescence change after 4-hr amplification in the DNAzyme system. (a) The fluorescence changes after 4-hour incubation in the presence of different concentrations of thrombin with the DNAzyme system. (b) Signals were linearly fitted by plotting the x-axis on a log scale (0.5-30 nM). Data are presented as mean values with standard deviations (error bars) derived from three independent experiments. Those error bars shorter than the height of the symbol are not shown in the graph. ***P* = 0.0052, ****P* = 0.0001 (unpaired t test, two-tailed *P* value, n = 3). LOD was defined as the lowest quantity of thrombin that produces a fluorescence signal $3\sigma_b$ higher than the average level of the blank sample. The calculated LOD of thrombin was ~0.36 nM. Source data are provided as a Source Data file.
